# Incomplete Follow-Up and Competing Risks as Sources of Bias in Vascular Surgical Investigations

**DOI:** 10.3390/jcm14207419

**Published:** 2025-10-21

**Authors:** Andrej Udelnow, Semion Smorodin, Efim Sinicin, Joerg Tautenhahn, Joerg Herold, Udo Barth, Zuhir Halloul

**Affiliations:** 1 Department of Vascular and Endovascular Surgery, Brandenburg University Hospital, Brandenburg Medical School “Theodor Fontane”, 14776 Brandenburg, Germany; joerg.tautenhahn@uk-brandenburg.de; 2 Department of Cardiology, Angiology, Pneumology, Nephrology and Intensive Care Medicine, Municipal Hospital Hanau, 63450 Hanau, Germany; samy.2709@gmail.com; 3 Department of Ophthalmology, Medical School of Hannover, 30625 Hannover, Germany; e.sinicin@yahoo.de; 4 Department of Angiologie, Kerckhoff Clinic, 61231 Bad Nauheim, Germany; joerg_herold@hotmail.com; 5 Division of Vascular Surgery, Department of General, Abdominal, Vascular and Transplant Surgery, Otto-von-Guericke-University Magdeburg, 39120 Magdeburg, Germany; udo.barth@med.ovgu.de (U.B.); zuhir.halloul@med.ovgu.de (Z.H.)

**Keywords:** vascular surgery, peripheral artery disease, incomplete follow-up, competing risk, Fine–Gray Model

## Abstract

**Background/Objectives**: The aim of this study was to determine whether incomplete follow-up and competing event risks may be associated with the clinical course after treatment and with known risk factors, such as chronic limb-threatening ischemia (CLTI), in peripheral artery disease (PAD) patients. **Methods**: Patients hospitalized with PAD who were treated by endovascular or open-surgical means and followed up were included in this retrospective observational study. The primary outcome was reintervention-free survival (RFS); the secondary outcomes and competing events were major amputation and death. The follow-up index (FUI), defined as the ratio between the real and the maximal follow-up interval, was determined for each patient. **Results**: The FUI depended significantly on the disease stage of CLTI (estimate: −0.16; *p*: 0.003), endovascular (0.17; *p*: 0.007) or open-surgical intervention (0.21; *p*: 0.007) and intra-hospital re-operation (−0.29; *p*: 0.002) and tended to decrease with age (−0.004; *p*: 0.09). Independent of disease stage, patients with claudication or CLTI with an FUI < 0.5 had shorter RFS than patients with a FUI ≥ 0.5 (Cox regression, *p*: 0.07; log-rank test, *p*: 0.03). When both the FUI and competing risks were considered using Fine–Gray regression analysis, CLTI was associated with RFS (*p*: 0.016), while FUI (*p*: 0.004), CLTI (*p* < 0.001), and the involvement of common femoral (*p* < 0.001) and posterior tibial arteries (*p* < 0.001) were associated with major amputation-free survival. **Conclusions**: Incomplete follow-up is associated with advanced PAD and may itself mask a worse outcome, such as reintervention, restenosis, major amputation, or death. Competing events should also be considered potential sources of bias. Therefore, the FUI and competing events should be reported, and conclusions should be drawn cautiously in both observational and randomized prospective clinical studies.

## 1. Introduction

Clinical trials investigating peripheral artery disease (PAD) or comparing new treatment methods focus on mid- and long-term outcomes, using overall survival, major amputation-free survival, or vessel patency as censored response variables. In most clinical trials, follow-up remains incomplete because patients discontinue participation or events other than the primary outcome occur.

Von Allmen et al. demonstrated that incomplete follow-up led to a profound underestimation of deaths and introduced the follow-up index (FUI) to quantify the completeness of follow-up.

FUI = (C − A)/(B − A),(1)

where C − A represents the follow-up period covered by this study, and B − A is the potential duration of follow-up until study closure [1].

An FUI of 1 indicates complete follow-up. It was supposed that a mean FUI differing from 1 by 0.1 may correspond to a 10% underestimation of mortality [1]. Since the competing risks may be subject to different cause-specific hazards, the Fine–Gray model describes the various effects of co-variates on the sub-distribution hazard function [2]. Other potential bias sources in survival analysis include clustering and time-variable hazards.

The aim of the present study was to evaluate the dependencies between incomplete follow-up and competing risks from various co-variates in a univariate comparison, followed by a regression analysis of potential clinical predictors and determinants of the clinical course after endovascular and/or vascular surgical intervention in PAD patients. Death, major amputation, reintervention and restenosis/re-occlusion were considered competing outcomes, while vessel patency and clinical co-factors served as predictors. In other words, anatomical and clinical co-factors were analyzed for their contribution to long-term event-free survival, considering the role of confounding effects on incomplete follow-up and competing risks.

## 2. Materials and Methods

### 2.1. Study Design and Inclusion Criteria

The investigation was conducted as a retrospective, observational, single-center study in a consecutive patient cohort with long-term follow-up. The treatment indications and principles were based on applicable German guidelines of the Association of the Scientific Medical Societies (AWMF) for PAD [3,4,5,6,7,8]. All patients hospitalized and treated between January 2012 and December 2014 were included if they had been admitted for the first time as elective or urgent inpatients suffering from PAD Fontaine stages II b–IV. The patients were followed up until 31 December 2016. Patients without an event were censored after the last visit. Patients with a history of lower-extremity treatment by surgery or intervention (including prior revascularizations by bypass or stent implantation, vascular events such as embolisms of cardiogenic and/or aneurysmal origin, acute occlusion/acute Leriche syndrome, aneurysms of the aorta or lower extremities and previous amputations), other than percutaneous angioplasty, were excluded from this study.

### 2.2. Clinical Data Assessment

Data collection was performed by accessing the electronic patient records of the hospital information system (Medico, Compu Group Medical SE & Co. KGaA, Koblenz, Germany, 2020). The imaging data (MRA, DSA, Doppler ultrasonography) of the interventions (percutaneous transluminal angioplasty with or without stenting) were recorded in the picture archiving and communication system (PACS). In addition to the assessed Doppler parameters, the following data were extracted from the medical records: Fontaine stage and diabetes type I or II, admission and discharge dates, demographic data, treatment methods and dates, reinterventions (occlusion or restenosis of the vessels of the treated lower extremity with re-operation or angiography with or without intervention), major amputations and death. Follow-up was pursued and documented by the clinical dispensary until 31 December 2016 every six months for the first year and yearly afterwards.

Urgent re-admissions due to clinical deterioration were also recorded. Patients without event occurrence were censored.

### 2.3. Ethics

According to the legal requirements of the district of Saxony-Anhalt in Germany, approval by the local ethics committee was not necessary for this noninvasive retrospective study, which used data from routine diagnostics. However, the data safety guidelines of Magdeburg University and Saxony-Anhalt were strictly followed. The ethical guidelines of the Helsinki Declaration of 2013 and the STROBE criteria for observational studies were fulfilled [9,10]. All patient records were pseudonymized, and the study’s data security standards complied with the requirements of the Saxony-Anhalt district, the Federal Republic of Germany and the European Union.

### 2.4. Statistical Analysis

Data analysis was performed using R 4.51. software [11]. Kaplan–Meier curves (KMCs) were calculated using the “survival” package (version 3.8-3) [12], and RFS was compared with the log-rank test. The “lattice” package (version 0.22-7) was used for multipanel diagrams [13], the “cmprsk” package (version 2.2-12) for the Fine–Gray-model calculations [14].

## 3. Results

### 3.1. The Completeness of Follow-Up Depended on the Fontaine Stage

**Figure 1:**
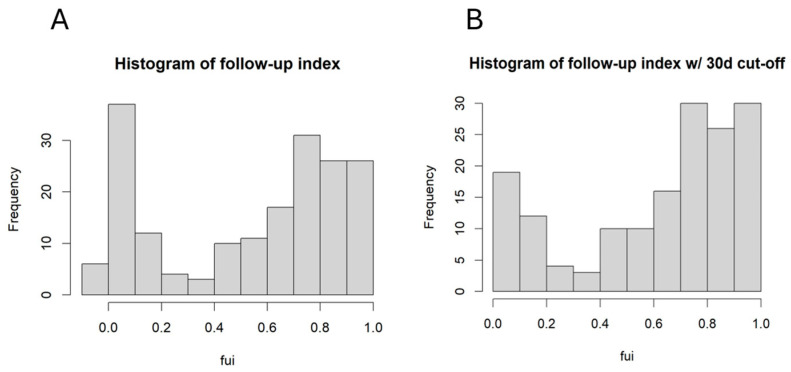
(**A**) Histogram of FUI for all patients; (**B**) histogram of FUI after excluding all patients with follow-up < 30 days.

Overall, 183 patients were included. One of the aims was to identify confounding factors for incomplete follow-up. The FUI was calculated for each patient, and the corresponding histogram is shown in Figure 1A.

**Figure 2:**
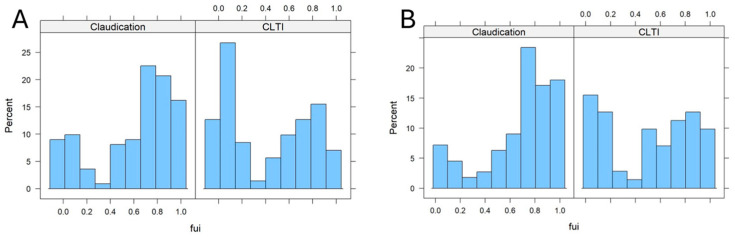
Histogram of the follow-up index in patients with intermittent claudication ((**left**) panel) and chronic limb threatening ischemia (CLTI, (**right**) panel). (**A**) For all patients; (**B**) patients with 30-day cut-off.

A large fraction of patients had a very low FUI. After excluding all patients with a follow-up < 30 days from further analysis, the distribution remained dichotomic, with a mean FUI of 0.61. Further investigation focused on factors influencing the FUI, as these factors may have contributed to additional bias. Figure 2 shows the FUI histograms for patients with different PAD stages (claudication [Fontaine stage 2] vs. chronic limb threatening ischemia [CLTI, Fontaine stages 3 and 4]).

The FUIs in the 30-day cut-off subcohort were markedly shifted to higher values in patients with claudication compared to those with CLTI (0.68 vs. 0.51; *p*: 0.004, Wilcox test; *p*: 0.002, *t*-test; see Figure 2B). This difference was even more pronounced when all patients were included (Figure 2A, without 30-day cut-off). These findings indicate a possible confounding bias of the PAD stage on the outcome results, as death and other adverse event rates may have been significantly underestimated in later compared to earlier stages. Patients with CLTI were obviously lost to follow-up earlier than those with earlier disease stages. Other independent co-factors were identified via univariate comparison between subcohorts with FUI< 0.5 vs. FUI ≥ 0.5 (Table 1) and multiple regression analysis of the FUI (see Table 2).

**Figure 3:**
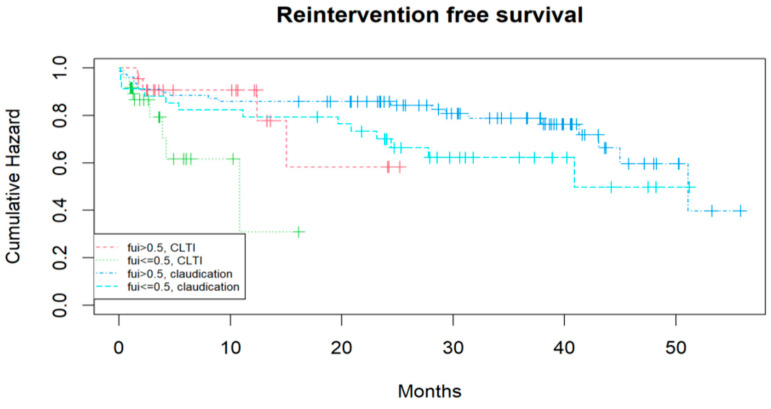
Kaplan–Meier curves of subcohorts with different PAD stages (claudication vs. CLTI) and different FUIs (>0.5 vs. ≤0.5) for reintervention-free survival.

**Figure 4:**
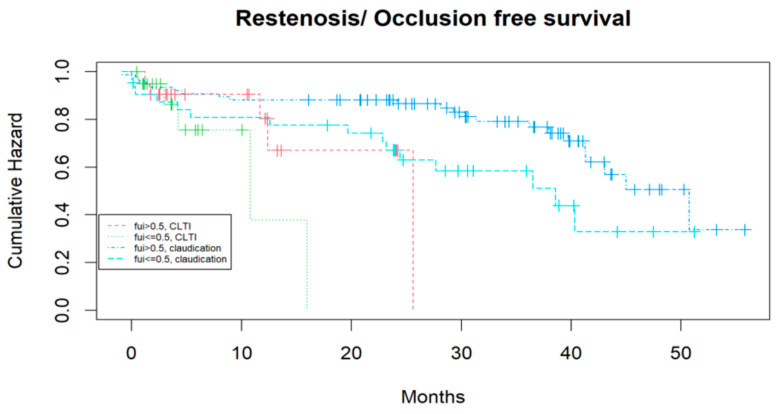
Kaplan–Meier curves of subcohorts with different PAD stages (claudication vs. CLTI) and different FUIs (>0.5 vs. ≤0.5) for restenosis- or occlusion-free survival.

Table 1 demonstrates a tendency toward a higher mean age in the subcohort with FUI < 0.05, while other co-factors did not differ significantly. The number of re-operations (including patients who first underwent endovascular treatment) was significantly higher in the FUI < 0.5 subcohort. The regression analysis (Table 2) identified the PAD stage (CLTI vs. IC) and intra-hospital re-operation as significant predictors; age showed a negative correlation, while endovascular or open surgical intervention (vs. best medical treatment) correlated positively with the FUI. This means that, the older the patient, the more the disease progresses; moreover, in cases with postoperative complications demanding surgical revisions, there is a higher risk of follow-up loss. Conversely, endovascular or open-surgical treatments were associated with a greater likelihood of continuous follow-up than conservative treatment. The effect of incomplete follow-up on event-free survival, independent of Fontaine stages, is illustrated with Kaplan–Meier curves for claudication vs. CLTI and FUI > 0.5 vs. ≤ 0.5 in Figure 3 and Figure 4.

CLTI patients had a shorter reintervention-free survival than those with claudication, and patients with FUI > 0.5 tended to have longer reintervention-free survival than those with FUI ≤ 0.5 (Cox regression, *p*: 0.07; log-rank test, *p*: 0.03). Similar results were observed for “restenosis- or occlusion-“free survival (Cox regression, *p*: 0.03; log-rank test, 0.01). One could argue that the shorter event-free survival was directly caused by the lower FUI if the follow-up had ended at that point; however, follow-up continued after these events until death, loss of follow-up, or study completion. Major amputation was also analyzed as a competing event in relation to the FUI, but because of the small number of events, no significant differences were detected (*p*: 0.08).

In summary, the completeness of follow-up was significantly associated with the PAD stage and showed a trend with age. Independently of PAD stage, event-free survival for restenosis/occlusion was significantly shorter in patients with shorter follow-up and tended to be shorter when reintervention was assessed. These findings point to a confounding bias caused by incomplete follow-up.

### 3.2. Identifying Prognostic Factors for the Mid-Term Outcome After Treatment Considering Competing Risks

**Figure 5:**
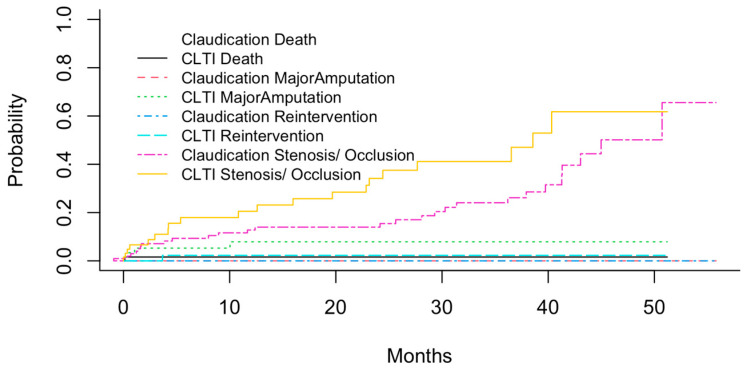
Kaplan–Meier curves showing the cumulative probabilities of competing events. Some curves are not visible because no events occurred within the subcohorts.

Apart from incomplete follow-up, competing events such as death, which may preclude other outcomes like reintervention or major amputation, must also be considered in the survival analysis. Figure 5 shows the cumulative probabilities of these different events (stenosis/occlusion, reintervention, major amputation and death) across different PAD stages, calculated using the cuminc function of the cmprisk R package (version 2.2-12). Overall, 46 reinterventions and 8 major amputations were necessary during this study, while 5 patients died.

As expected, the probabilities of all events were higher in patients with CLTI compared to those with claudication. The highest probabilities were observed for stenosis/occlusion, with up to 50% of CLTI patients suffering from new or restenosis within 4–5 years after initial treatment. Other events were less common, with major amputations being the most frequent in CLTI patients. However, it is known that the cumulative total failure probability (defining all events as adverse or treatment “failure”) may exceed 1, which statistically impossible [15]. Therefore, the associations between co-factors and prognosis have to be calculated using the Fine–Gray sub-distribution hazard model.

Therefore, a co-variance matrix was built from potential risk factors, including the FUI, age and postoperative vascular anatomy, and a competing risk regression on reintervention-free survival was performed based on the Fine–Gray model in the cmprsk R package. The results are listed in Table 3.

Only PAD stage (significantly) and anterior tibial artery stenosis (tendentially) contributed to the risk of reintervention. The same regression analysis was performed on major amputation-free survival, and the results are listed in Table 4.

Besides the FUI and PAD stage, stenosis/occlusion of the common femoral and posterior tibial arteries contributed significantly to the risk of major amputation, with the FUI showing a protective effect. While these regression analyses accounted for competing risks, it cannot be stated with certainty that one vessel was more important than another in limb preservation. However, it appears plausible that the common femoral artery plays a key role in the further course of the disease. In summary, the post-treatment clinical course was characterized by time-dependent competing events that had to be considered when assessing event-free survival. Both reintervention-free survival and the major amputation-free survival were mainly determined by the PAD stage at admission; however, the latter was also influenced by the FUI and the condition of the common femoral and posterior tibial arteries.

## 4. Discussion

The present investigation revealed a strong association between the FUI and PAD stage (claudication vs. CLTI) (see the multiple regression results in Table 2). Among other factors, such as endovascular or open-surgical intervention, age and intra-hospital re-operation, the PAD stage contributed to a higher risk of early follow-up loss and should therefore be considered an important source of confounding bias in clinical studies. Reintervention-free survival and stenosis-/occlusion-free survival depended on the PAD stage and FUI when competing risks were not explicitly considered. However, even after recalculating the sub-distribution hazards using the Fine–Gray model, the PAD stage remained a significant contributor for the reintervention-free survival, and both the PAD stage and FUI contributed significantly to the major amputation hazard. This means that, for this observational study, both the FUI and competing events represented considerable sources of confounding bias in the outcome analysis. This bias can be reduced by applying the Fine–Gray model and including the FUI as a prognostic factor. Few clinical investigations have considered these bias sources. A PubMed database search using the keyword “fine-gray competing risk” and the filters “Clinical Trial, Randomized Controlled Trial” identified only 27 studies (on 17 August 2025, 1 a.m.), none of which considered vascular diseases. While confounding bias is well recognized in observational studies and should always be accounted for, for instance, by adhering to the STROBE guidelines [9,10], incomplete follow-up and competing risks have rarely been addressed in prospective randomized studies, perhaps because randomization seemed to balance even those co-factors that are not randomized.

The BEST-CLI trial, a large prospective randomized trial comparing a surgery-first approach versus an endovascular-first approach for femoropopliteal reconstruction, reported that 786 of the 1847 initially randomized patients completed the follow-up, while 553 patients died or were lost to follow-up from other reasons [16].

In the BASIL trial, another comparative investigation focusing on below-the-knee reconstructions, 241 of 345 enrolled patients reached the 24-month follow-up, while the remainder were lost to follow-up from various reasons, with higher mortality in the venous bypass group [17]. The latter finding led the authors to conclude that the superiority of endovascular-first compared to bypass-first was mainly due to the excess mortality in the bypass group. This excess mortality was believed to be mainly cardiac, though the underlying reason was not established [18].

In both trials, death was registered as a secondary outcome, but as a competing event, it precluded further assessment of the primary outcome: primary or secondary vessel patency. As incomplete follow-up and competing risks are not necessarily evenly distributed across subcohorts, the present study investigated the confounding effects.

However, as is obvious in the BASIL-2 trial [17], there may be unidentified factors that substantially increase the risk of adverse events, such as death, and contribute to confounding bias. In fact, as was demonstrated in the present investigation, patients of advanced age and higher PAD stages could not be adequately followed up, and in this subcohort, the number of un-registered adverse events, such as major amputation, stroke, myocardial infarction and cardiovascular death, may have been, and probably was, roughly underestimated. Moreover, a subgroup of patients with rapidly progressing multilocal vascular stenosis and chronic ischemia may also have been insufficiently followed up due to rapid deterioration. However, this remains speculative until the respective risk factors and causal relationships are identified. Time-varying co-factors, such as disease stage and secondary interventions, cannot be controlled in randomized trials and may influence the results or depend on various factors.

One of the most relevant limitations of prospective randomized trials contributing to the above-mentioned effects involves the inclusion and exclusion criteria, which tend to exclude older, multimorbid patients with advanced disease and the highest risk of adverse outcomes [19]. The main limitation of the present investigation, aside from the lack of randomization, was its selective inclusion criteria: patients with prior interventions altering the vascular anatomy, such as stent implantation or bypass surgery, were excluded to limit cohort heterogeneity. This may restrict the study’s representativeness regarding prognostic assumptions. However, since one of the study’s aims was to identify co-factors (i.e., the states of native vessels) which could contribute to different event hazards during follow-up, this limitation was necessary to reduce anatomical complexity. Previous interventions, including bypasses, stents and patches, may have substantially changed the anatomy of the leg and event hazards. Therefore, the present study should be classified as a preliminary or “pilot” investigation.

However, the results shown above demonstrate the key aspects of confounding bias when performing a study and analyzing longitudinal data.

The follow-up index and the competing risk analysis based on the Fine–Gray model describe different sources of bias; however, there is still no established mathematical model or algorithm which unifies incomplete follow-up, competing risks, clustering and time-variant co-factors into a unified framework. Therefore, in this study, the FUI was included as a co-factor in the Fine–Gray model to account for incomplete follow-up bias as a possible prognostic risk factor. Conversely, it is not possible to include a competing risk analysis conducted using the Fine–Gray model into the Kaplan–Meier curves of the subcohorts with different Fontaine stages and FUIs (<0.5 vs. ≥0.5), as the Fine–Gray model primarily addresses the problem of the cumulative incidences > 1 in competing risks contexts. The model can accurately predict the direction (negative or positive contribution) and the level of significance (*p*-value) but does not provide a reliable quantitative value of the estimate [15]. While the development of a model unifying all four bias sources would be desirable, this would at minimum demand the complete follow-up of one cohort in comparison with an incomplete follow-up of the same cohort under standard follow-up conditions. Future research, such as comparative analyses between endovascular and open-surgical treatments within clinical registries or randomized prospective studies, should consider the sources of bias discussed above (incomplete follow-up, competing risks, clustering and time-dependent risk factors) as well as contributing factors (age, Fontaine stage, re-operation and type of treatment). It would be valuable to define a metric, similar to the FUI, that can quantitatively estimate the degree of underestimation of a (competing) event and its implications for subsequent statistical analyses.

## Figures and Tables

**Table 1:** Comparison of subcohorts with FUI < 0.5 vs. FUI ≥ 0.5. Abbr. DM: diabetes mellitus; CIHD: chronic ischemic heart disease; RI: renal insufficiency; §: *t*-test; #: chi-square test; * significant.

Co-Factor	All (N = 183)	FUI ≥ 0.5 (N = 112)	FUI < 0.5 (N = 48)	*p*
Age (years)	69	67	71	0.09 §
Sex = female (%)	33	34	38	0.88 #
DM (%)	43	44	46	0.95 #
CIHD (%)	34	33	38	0.97 #
RI w/o dialysis (%)	14	13	13	0.72 #
RI w/ dialysis	6	5	8	1 #
Endovascular (%)	60	65	50	0.1 #
Open surgical (%)	27	26	27	1 #
Emergency surgery (%)	7	8	0	0.88 #
Re-operation during hospital stay	7.6	4.5	18.8	0.008 # *
Bleeding	10	10	6	0.8#
Major amputation within 12 months	0.8	2.7	21.4	0.03 # *
Death within 12 months	0.8	0.9	0	1 #

**Table 2:** Results of the multiple regression analysis on FUI, listing the initial and final models. Signif. codes: 0.001 ‘**’; 0.01 ‘*’.

Co-Factor	Initial Model		Final Model	
	Estimate	*p*	Estimate	*p*
(Intercept)	0.78	<0.001	0.79	<0.001 **
Age	−0.004	0.08	−0.004	0.09
Female sex	0.06	0.27		
Endovascular	0.17	0.005 *	0.16	0.007 **
Open surgery	0.21	0.005 *	0.20	0.007 **
CLTI	−0.12	0.02 *	−0.12	0.026 *
Intra-hospital re-operation	−0.29	0.003 *	−0.29	0.002 **

**Table 3:** Results of the competing risk regression analysis for reintervention-free survival. Vessel-related co-factors indicate arteries that were high-grade stenosed or occluded. Abbreviations: CI: confidence interval; Coeff: coefficient. * significance tendency.

Co-Factor	Exp (Coeff)	2.5% CI	97.5% CI	*p*
Age	0.983	0.95	1.02	0.31
FUI	0.47	0.15	1.53	0.21
CLTI	2.32	1.18	4.55	0.015 *
Intervention	1.20	0.56	2.56	0.64
Profound femoral artery	0.78	0.25	2.49	0.68
Common femoral artery	0.48	0.17	1.34	0.16
Spf. femoral artery	0.57	0.23	1.41	0.23
Fibular artery	1.52	0.71	3.23	0.28
Anterior tibial artery	0.48	0.23	0.99	0.05.
Posterior tibial artery	1.22	0.60	2.48	0.57

**Table 4:** Results of the competing risk analysis on major amputation-free survival. Further explanations and abbreviations are provided in Table 2. ** highly significant (*p* < 0.01).

Co-Factor	Exp (Coeff)	2.5% CI	97.5% CI	*p*
Age	0.94	0.85	1.04	0.24
FUI	0.015	8.28 × 10^−4^	0.265	0.004 **
CLTI	5.2 × 10^7^	8.47 × 10^6^	3.21 × 10^8^	<0.001 **
Intervention	2.44	0.63	9.45	0.2
Profound femoral artery	0.15	0.008	2.57	0.19
Common femoral artery	3.74 × 10^7^	2.72 × 10^6^	5.14 × 10^8^	<0.001 **
Spf. femoral artery	1.12	0.27	4.67	0.88
Fibular artery	0.19	0.24	1.46	0.11
Anterior tibial artery	0.75	0.16	3.57	0.72
Posterior tibial artery	2.16 × 10^7^	2.49 × 10^6^	1.88 × 10^8^	<0.001 **

## Data Availability

The data are not publicly available due to ethical concerns but anonymized data may be provided by the authors for scientific projects or meta-analysis.

## Abbreviations

The following abbreviations are used in this manuscript:

CLTI	chronic limb-threatening ischemia
RFS	reintervention-free survival
FUI	follow-up index
PAD	peripheral artery disease
MRA	magnetic resonance angiography
DSA	digital subtraction angiography
KMC	Kaplan–Meier curves
IC	intermittent claudication
CIHD	chronic ischemic heart disease
Coeff	coefficient
